# Supplementary material for: Gonadotropin Releasing Hormone (GnRH) Triggers Neurogenesis in the Hypothalamus of Adult Zebrafish
Source: Int J Mol Sci. 2021 May 31;22(11):5926. doi: 10.3390/ijms22115926 (PMC8198740; doi:10.3390/ijms22115926)
Supplement: Supplementary file 1 [file ijms-22-05926-s001.zip › ijms-1195081-supplementary.pdf]

SUPPLEMENTAL FIGURES
Ceriani and Whitlock (ijms-1195081)

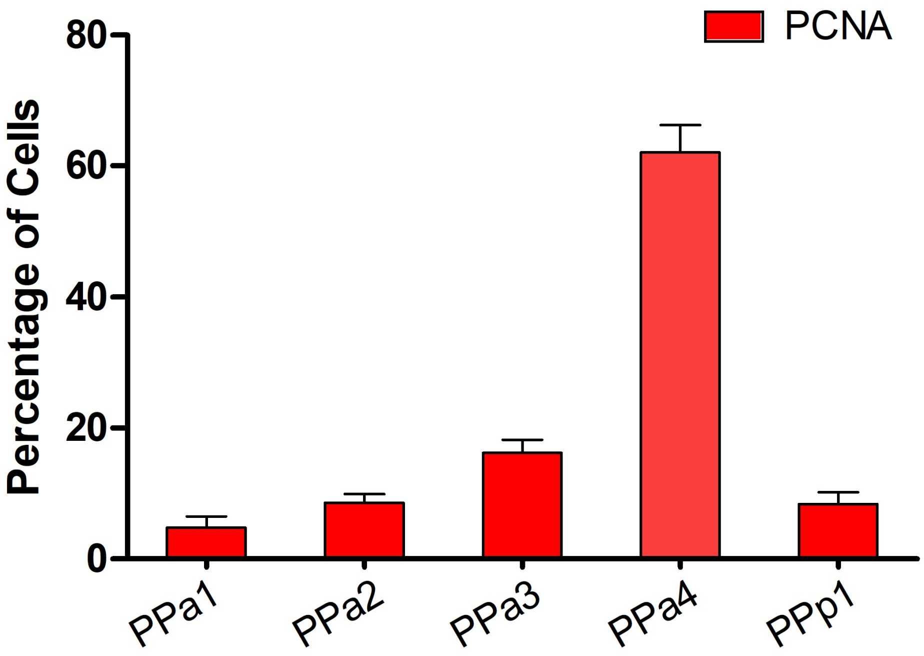

Supplemental Figure S1. Quantification of the distribution of PCNA+ cells in the POA. Percentage of PCNA+ cells located in representative sections of the POA (PPa1, PPa2, PPa3, PPa4 and PPp1). For each representative section of the POA, a number of transverse paraffin sections (PPa1 = 4, PPa2 = 8, PPa3 = 10, PPa4 = 14 and PPp1 = 4) of 5 µm were obtained and the PCNA+ cells were counted.

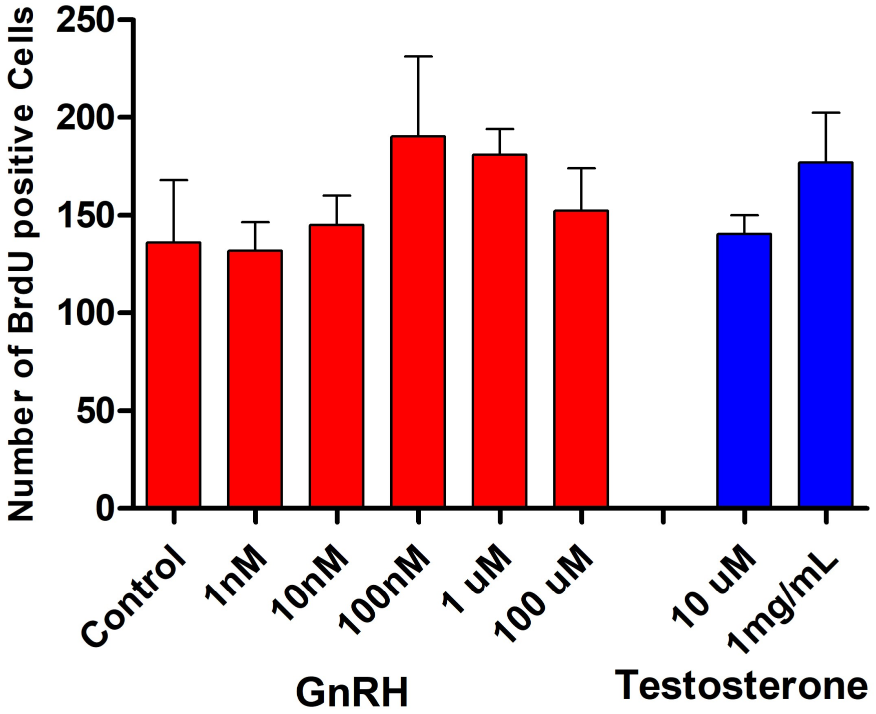

Supplemental Figure S2. Determination of optimal GnRH and testosterone concentration. Optimal hormone concentration was determined according to the increase of number of $BrdU^+$ cells in treated versus control fish. Three fish were used for each treatment. The BrdU+ cells were counted in cryosections of 20 μm of the POA (10 sections).
